# Caregivers’ concerns about the sexual and reproductive health of women with intellectual disability in Iran: a qualitative study

**DOI:** 10.1186/s12978-024-01765-6

**Published:** 2024-03-12

**Authors:** Ziba Taghizadeh, Maryam Farmahini Farahani, Malihe Nourollahpour Shiadeh, Kowsar Qaderi

**Affiliations:** 1 Reproductive Health and Midwifery Department, Nursing and Midwifery Care Research Center, School of Nursing & Midwifery, Tehran University of Medical Sciences, Tehran, Iran; 2 Department of Midwifery, Faculty of Nursing and Midwifery, Tehran Medical Science, Islamic Azad University, Tehran, Iran; 3 Sexual and Reproductive Health Research Center, Mazandaran University of Medical Sciences, Sari, Iran; 4 Midwifery Department, School of Nursing and Midwifery, Kermanshah University of Medical Sciences, Kermanshah, Iran

**Keywords:** Sexual and reproductive health, Intellectual disability, Caregivers

## Abstract

**Background:**

Women with intellectual disability (ID) have many sexual and reproductive problems. This study was conducted to explain the sexual and reproductive health considerations of women with ID from the perspective of their caregivers in a qualitative approach.

**Methods:**

This study was a qualitative research conducted with a content analysis approach in Iran. The sampling method used was targeted sampling with maximum possible variation, which was continued until data saturation. For data collection, in-depth and semi-structured interviews were conducted with 21 participants, including 8 mothers, 6 caregivers, and 7 specialist caregivers who had experience working with women with intellectual disabilities. Data analysis was conducted using the conventional content analysis method proposed by Zhang and Wildemuth.

**Results:**

Two main themes, four categories and 12 subcategories emerged from the data analysis. The themes include “Reproductive health concerns” and” "Sexual health concerns”. This means that this group of women has many problems with menstrual hygiene and vaginal infections. On the other hand, caregivers were concerned about the manifestations of unconventional sexual behaviors and difficulties in controlling sexual behaviors as well as the risk of sexual abuse.

**Conclusion:**

The results of the present study show that it is not only necessary to provide women with ID with practical instructions on menstrual hygiene and sexual self-care, but also that regular examinations of the reproductive system by obstetricians or midwives, especially in care centers, seem essential.

## Abstract (plain language summary)

Women with intellectual disability, experience many sexual and reproductive problems. The results of previous studies have highlighted the problems related to menstrual hygiene, contraceptive choice, diagnosis and treatment of sexually transmitted diseases, and cancer screening among women with ID. women with intellectual disability are also more exposed to the risk of sexual abuse. In Iran, there are few studies on the sexual and reproductive health of this group, and there is little information in this regard. Therefore, we decided to conduct a study to investigate the sexual and reproductive problems of women with intellectual disability. For this purpose,we interviewed 21 mothers and caregivers who were directly responsible for the care of women with intellectual disability. The results of the study showed that women with intellectual disability have many problems related to menstrual hygiene and vaginal infections. Caregivers were also concerned about inappropriate sexual behaviors among women with intellectual disability and sexual abuse of these women. According to the caregivers, practical training of women with intellectual disability in menstrual hygiene and sexual self-care can help to reduce their sexual and reproductive problems. This training should be visual and repeated. To prevent vaginal infections, regular genital examinations by obstetricians or midwives are also important, especially in care centers.

## Background

The phenomenon of intellectual disability (ID) has a long history that is as old as humanity itself. Throughout this eventful history, people with ID have been familiar with a label. In the past, people with ID were considered asexual or unable to have a sexual life [1]. Consequently, the sexual desire of people with ID was often unrecognized and suppressed, and when expressed, it was introduced as something abnormal and labeled as hypersexual or deviant [2]. With the economic and political development of societies and the establishment of institutions and associations for people with disability since the early 1980s, a new movement regarding people with disability began, and the perspective of social rehabilitation replaced the previous ideas. In this social model, people with disability have the right to gain equal opportunities and rights as other members of society through social interaction in all personal and social aspects [3]. According to the World Health Organization, sexual and reproductive health is one of the fundamental dimensions of health that improves a person’s life, and people with disabilities, as human beings, have the same sexual and reproductive rights as other members of society [1].

Women with ID face numerous sexual and reproductive challenges. The results of previous relevant studies have highlighted the problems related to menstrual hygiene, contraceptive choice, diagnosis and treatment of sexually transmitted diseases and cancer screening among women in this group [4]. Women with ID are also more at risk of sexual abuse, with the literature review showing that one in three people with ID will be sexually abused [5, 6]. However, it is impossible to estimate the exact prevalence of sexual abuse among people with ID because they are unable to report sexual abuse [7]. Unfortunately, there are no statistics or reports on sexual abuse of people with ID in Iran, as it is considered extremely taboo by the public and even by the authorities and health care providers.

The challenges faced by women with disabilities in their sexual and reproductive lives are not necessarily due to their disability, but may also be due to a lack of attention and legal and social support [8, 9]. There are few cases of women with ID who have not been physically, emotionally or sexually abused, forcibly married or forcibly sterilized [10].

Meeting the sexual and reproductive needs of women with ID depends mainly on the performance of their caregivers and parents, and undoubtedly their views on this issue will have a decisive influence on the quality of sexual and reproductive health services for these people [11, 12]. Negative attitudes of people, especially health workers and parents of women with ID, towards sexual and reproductive issues of people with ID play a more important role in hindering their access to sexual and reproductive health services than their disability [4]. It is evident from the studies that despite the increasing acceptance and knowledge of society, especially among caregivers, about the dimensions of sexuality in women with ID, some aspects of sexuality are still controversial in this group. This issue may vary according to the cultural, moral and religious conditions of each society [13, 14].

In Iran, there are few studies on sexual and reproductive health of this group, and there is limited information in this regard, so it is necessary to conduct qualitative research that examines this concept in detail. Due to the direct, enormous and proven influence of the perspective of parents and caregivers of women with ID on the fulfilment of their sexual and reproductive needs, the researcher decided to explain the sexual and reproductive health considerations of women with ID from the perspective of their caregivers in a qualitative approach. This information can help health authorities revise current laws and policies and develop new approaches to the sexual and reproductive health of women with ID.

## Methods

This study was a qualitative study. Participants were selected using targeted sampling to capture as many diverse opinions and a broad range of perspectives on the phenomenon as possible. Participants included caregivers with at least three years of work experience with women with ID and parents of women with ID aged 15–45 who were directly responsible for their care. Participants were also literate and spoke Persian. The researchers approached the care centers and day rehabilitation centers affiliated with the welfare organization to find the first participants. Subsequent participants were selected based on the results of the continuous comparative analysis and the recommendations of the initial participants, taking into account as much diversity as possible in terms of age, education and work experience Sampling continued until data saturation was reached. This means that sampling continued until no more new information was obtained and the data was repeated. Interviews were conducted after obtaining approval from the Ethics Committee of Tehran University of Medical Sciences and permission from Tehran Welfare Organization. In-depth and semi-structured interviews were conducted to collect data. Prior to the interviews, the objectives, methodology, voluntary participation and confidentiality of their statements were explained to the participants. The participants' verbal and written consent was also obtained for the recording of the interviews. Each interview was conducted individually and with respect for the privacy of the participants. The time and place of the interview were determined according to the participants' opinions. The interviews lasted between 35 and 90 min (average: 60 min). While longer interviews can contribute to a greater richness of data, the interviewers focused more on the quality of the interview and achieving saturation than on the desired length. Interviews began with guiding questions and were then guided by exploratory questions. Open-ended follow-up questions were used to obtain detailed descriptions. Some of the guiding questions were as follows:

“Please tell me about your experiences with issues related to the sexual and reproductive health of women with ID.”

“Describe your experiences with your child (client) during puberty.”

“What are the issues faced by women with ID during puberty?”

Exploratory questions such as “Please tell me more about it,” were also used to increase the depth of the interviews.

To ensure the credibility of the data, the interviews were transcribed by the researchers immediately after recording. The non-verbal messages of the participants, such as the tone of voice, silence and crying recorded during the interview, were added to the transcripts, as there are some concepts and patterns hidden in the data that need to be extracted. In addition, some of the transcripts of the interviews were sent back to the participants to confirm the accuracy of the data. After the participants gave their feedback, the concepts were modified and the interpretations were adjusted based on their input. (Member Checking) and the transcripts of the interviews were reviewed through peer debriefing. In this way, the researchers shared and discussed their findings, interpretations and analyzes with qualified peers. The purpose was to address potential biases, recognize overlooked details, and improve the reliability of the research by obtaining feedback and insights from other researchers. This process helped researchers to ensure the coherence of their arguments and establish the trustworthiness of their research. MAXQDA10 software was used for the initial management of the qualitative data. It helps the researchers to organize, manage and code the qualitative data more efficiently. The collected data were analyzed using the content analysis method proposed by Zhang and Wildemuth [15]. In this way, the transcripts were read several times to extract hidden concepts from the data. The units of meaning were then defined and appropriate codes were written for each of them. Similar codes were assigned to the subcategories, and the subcategories were assigned to the main category based on their common characteristics and their relationship to each other. Then, hidden patterns were revealed and extracted into the themes.The researchers tried to avoid introducing their own preconceptions into the data collection and analysis, and spent enough time conducting in-depth interviews and analyzing them to ensure a deep and accurate understanding of the data. In addition, to increase the validity of the data, all stages of data analysis were reviewed and approved by members of the research team.

## Results

A total of 21 participants were interviewed, including eight mothers who had daughters with ID and cared for them, six caregivers from care centers for women with ID with at least three years of work experience, and seven health professionals with at least three years of experience working with women with ID, including a: gynecologist, a midwife, a psychologist, a general practitioner, a teacher, a professional trainer, and a manager of care centers for women with intellectual disabilities. The age range of the participants was between 28 and 62 years and the range of years of work experience of the caregivers was 3 to 26 years.The characteristics of the study participants are listed in Table 1.

**Table 1:** The demographic characteristics of the participants in the study

Experience work	Education	Age	Participant		
–	MS	50	1	Mothers	
–	Diploma	42	2		
–	Diploma	55	3		
–	Primary school	57	4		
–	Secondary school	45	5		
–	BS	50	6		
–	Associate degree	48	7		
–	Illiterate	62	8		
15	Diploma	40	9	Caregivers	
24	Diploma	42	10		
7	Diploma	53	11		
3	Secondary school	35	12		
19	Diploma	40	13		
4	Associate degree	34	14		
25	MS	51	15	Teacher	Specialists
5	PHD in gynecology	56	16	Gynecologist	
15	BS	42	17	Professional trainer	
18	PhD in psychology	44	18	Director of a center for the intellectual disabled	
10	MS	47	19	Psychologist	
11	MD	54	20	Physician	
3	BS	28	21	Midwife	

The data analysis revealed two main themes, four categories and 12 subcategories. The themes include “reproductive health concerns “ and “Sexual health concerns”. Table 2 shows the main themes and the extracted categories.

**Table 2:** The main themes and extracted categories

Themes	Categories	Subcategories
Reproductive health concerns	The challenges of menstruation	-Menstrual hygiene -Premenstrual mood swings -Different opinions about the occurrence of menstruation
	Vaginal infection	-Recurrent vaginal infections -Predisposing factors for vaginal infections -Prevention and treatment
Sexual health concerns	Sexual behaviors management	-Positive attitudes towards the sexual needs of women with ID -Uncertainties about sexual education for women with ID -Inappropriate sexual behaviors -Challenges related to managing the sexual behaviors of women with ID
	Sexual abuse	-The risk of sexual abuse -Unintended pregnancy

The first theme: “ Reproductive health concerns”.

One of the main concerns of caregivers was menstruation in women with ID. Most mothers were concerned about their daughter’s menstruation around the age of puberty. The worry of expressing menstrual issue in public and not being able to maintain menstrual hygiene became their nightmare.

“I am worried that when she menstruates, she announces it everywhere.” (Participant No. 3)

The caregivers of women with ID also pointed out mood swings before their menstruation. They felt that the symptoms of premenstrual syndrome, such as mood swings and cramps, manifest themselves in the form of intense emotional behaviors such as constant crying and shouting and sometimes convulsions, due to the inability to fully express and communicate pain symptoms and the low emotional tolerance threshold in women with ID.

"Some become aggressive and hurt themselves or others before menstruation.” (Participant No. 10).

The Caregivers had different opinions about the occurrence of menstruation in these women. Most believed that the use of menstrual suppression methods should not be performed on all women with ID, and that decisions should be made individually based on their cognitive abilities. For example, women who can manage their toilet hygiene can usually learn to manage their menstrual hygiene.

“In my opinion, it is unnecessary to do anything about menstrual suppression for women who can manage their toilet hygiene. But for women with very low intelligence, it is better to suppress menstruation and have a hysterectomy.” (Participant No. 20).

“In these centers, they learn to change their sanitary napkins on their own or with minimal help and observe menstrual hygiene.” (Participant No. 17)

However, some caregivers considered menstruation useless and annoying and believed that it should be suppressed.

Most mothers, on the other hand, considered menstruation useful and necessary for their daughters’ health and rejected the use of menstrual suppression methods.

“I have never tried to suppress their menstruation with medication because menstruation is good for women's health.” (Participant No. 8)

“It is better for her to menstruate. If her mind is sick, at least her body stays healthy.” (Participant No. 2)

According to the reports of the caregivers, the prevalence of vaginal infections in women with ID was also high. They believed that poor personal and menstrual hygiene in women with ID put them at risk of female infections, particularly urinary tract and vaginal yeast infections. This problem was observed more frequently in women with ID with lower intelligence levels. Moreover, since vaginal medications were not used for virgin women and most of them were still virgins, the treatment of vaginal infections was incomplete and led to frequent recurrence of the infection.

“Vaginal and urinary tract infections are more common in women who wear diapers due to incontinence." (Participant No. 3)

“ Since most of them are still virgins, a topical cream is used instead of vaginal cream.” (Participant No. 13)

The Caregivers pointed out the need to pay more attention to their personal and menstrual hygiene and to have routine examinations of their reproductive system by midwives or gynecologists.

The second theme: "Sexual health concerns''.

Although all caregivers acknowledged the presence of sexual desire in women with ID, they had different opinions on sexual education for these women. Most mothers took a more conservative approach to sexuality education and had doubts about this.

“In my opinion, it is better not to talk to them about this subject, because they might get carried away with sexual topics”. (Participant No. 2)

“I don’t want my child to understand any of these subjects and I’m afraid to give lessons in this area.” (Participant No. 5)

In contrast, most caregivers agreed with providing sexuality education but were unable and confused about how to teach women with ID in this regard.

“I am confused and would like an expert in this field to teach us how to deal with their inappropriate sexual behaviors.” (Participant No. 15)

“ Their physical problems can be managed with medication, but controlling her sexuality is a challenge. I really don’t know what to do. Sexual desire is like food. We can’t ignore it” (Participant No. 16).

Some caregivers believed that the cause of their inappropriate sexual behavior was the lack of emotional attention to them and the lack of opportunity to show their feelings.

“Most of them have been rejected by their families. Since they lack affection, they communicate to find affection. They caress each other, hug each other and kiss each other. Sexual behaviors may start after these emotional behaviors." (Participant No. 3).

In addition, according to the caregivers, the energy stored by their inactivity turns into sexual energy.

“We should consider sports activities for them and help them release their excess energy.” (Participant No. 19)

The Caregivers pointed out that women with ID have excessive sexual fantasies. They believed that the origin of this extreme fantasy in women with ID lies in the impossibility of real communication with the opposite sex. Most of them cannot communicate properly with others, especially with the opposite sex, because they are limited in social communication and lack social skills. This leads to inappropriate sexual behaviors in public, such as excessive intimacy, hugging or kissing strangers and certain sexual acts in public (masturbation). A small number of caregivers pointed out that their libido can be controlled by medication.

One of the most common concerns of caregivers, especially parents, was the risk of sexual abuse. Instances of sexual abuse reported by caregivers in this study included: sexually harassing phone calls, being shown pornographic photos and videos, unwanted touching of their private parts, and unwanted sexual relations. According to the caregivers, sexual abuse often takes place within the family and among relatives.

“Unfortunately, some of them are sexually abused by their relatives.” (Participant No. 18)

## Discussion

This study conducted in Iran, aimed to explain the concerns of caregivers of women with ID about their reproductive and sexual issues. According to the present research results, menstruation management in women with ID was one of the most challenging issues for caregivers. women with ID often depends on their caregivers for menstrual hygiene and change sanitary napkins. Wilbur (2022) also pointed out that menstrual management in women with ID became one of the difficult issues for their caregivers[16]. However, this problem was seen more frequently among women with severe intellectual disabilities. According to caregivers, premenstrual mood changes and dysmenorrhea, especially in those with moderate and severe ID, manifest as intense emotional behaviors such as constant screaming, shouting, and self-harm. Cooper (2019) stated that the incidence of these symptoms is higher in a group of people with ID who cannot express their feelings and report the symptoms of their disease [17].

However, most mothers believed in menstruation is useful and necessary for their daughter’s health. They disagreed with using methods for menstrual suppression. Also, except for a small group of caregivers, who considered menstruation to be useless, most of them believed that menstrual suppression in women with ID violates their human rights and were against it [18, 19]. According to the opinion of the American College of Nurse-Midwifery (ACNM), girls who can manage their toilet hygiene can usually learn to manage their menses[20]. The research results showed that education and support effectively promote self-management of menstrual hygiene in women with ID. Therefore, frequent visual education and practical exercises related to menstrual hygiene skills effectively and safely solve this problem [21].

In this study, caregivers pointed out that urinary tract infections are more common in women who wear diapers due to urinary and fecal incontinence. This seems to be due to their poor personal and menstrual hygiene. In addition, they are unable to report the symptoms of the disease. Therefore, they are not diagnosed with the disease until later. Keenan also stated that urinary and fecal incontinence is the most important risk factor for urinary and genital infections in women with ID [22]. According to this study, it is necessary to have routine examinations of their reproductive system by midwives or gynecologists.

Encouragingly, all caregivers acknowledged the presence of sexual desire in women with ID. They also felt that this need exists in all people with varying degrees of disability. But they had different views on sexual education for women. Most mothers had a rather conservative attitude towards sex education and had doubts. In contrast, most of the caregivers agreed to provide sexual education to these women. It is important to point out that even the caregivers who agreed to provide sexuality education to people with a disability, were unsure about how to do so.

Many previous studies have confirmed the findings of the present study [23–26]. Pownall cited feelings of confusion and uncertainty about how to teach sexual topics, as well as feelings of fear and embarrassment, as reasons for mothers’ reluctance to provide sexual education to their children with ID [27]. Maguire also stated that caregivers of people with ID express their inability to provide sexual education because there are no guidelines for sexual education [28].

In the present study, caregivers were concerned about inappropriate sexual behaviors among women with ID and their sexual abuse. Some caregivers felt that cultural taboos around sexuality were a barrier to sexual education for women with ID. Some caregivers considered the lack of emotional attention to them as the cause of unconventional and uncontrolled sexual behavior in women with ID. According to some caregivers, the intensification of their sexual desire is also due to the fact that their stored energy is not released due to their inactivity. Akrami believes that adolescents with ID are less likely to participate in games and daily activities and that there is no adequate and appropriate program for their leisure time. As a result, their energy is not released appropriately and manifests itself in the form of intense emotions, including intense sexual desire [29].

According to caregivers, emotional attention and affection from families toward them, sex education, especially self-care education, reducing sexual stimuli in their environment, exercise and physical activity to dissipate their energy, and sometimes prescribing medication to reduce sexual desire can control their sexual behavior.

It is important to point out that inappropriate sexual behavior can pose a risk for sexual abuse. Instances of sexual abuse reported by caregivers in this study included: sexually harassing phone calls, showing pornographic photos and videos, unwanted touching of their private parts, and unwanted sexual relations. According to the parents and caregivers, most of these abuses were committed by their family members because they were restricted in their social communication. The results of Pasha’s study confirm the findings of the present study. The results of this study showed that people with ID were sexually abused by their family members (brother, father or stepfather) or their acquaintances, neighbors or caregivers in about 89% of cases [30]. People with intellectual disabilities, due to their weak cognitive, social and verbal skills, their limited awareness of sexual issues and their lack of social opportunities to develop skills in this area, do not learn how to control and express their sexual desires based on human and social norms. Therefore, education aimed at developing emotional and social skills is necessary for people with ID and prevent them from being sexually abused [5, 31]. Since most people with ID have difficulties with abstract thinking, teaching should be simple, objective and delivered through multisensory stimuli. Visual instructions (video, coloring pictures), modeling and role-playing are useful methods for them. It is also important to provide positive reinforcement to promote retention of skills [32].

The present study had some limitations. Some parents did not want to participate in the study due to the sensitivity of the research topic and feeling embarrassed to talk about sex. Therefore, the researcher tried to reduce this limitation by explaining the importance of the study, establishing friendly communication and interaction with the participants, and taking enough time to adhere to ethical principles to gain the trust of the participants. In view of the topic being influenced by social and cultural factors and the nature of the qualitative research, the generalizability of the research results to other population groups and cultures is not guaranteed.

## Conclusion

In order to improve the sexual health of women with ID, it is necessary to design and implement programs for parents to change their attitudes towards sexual education for their children. Also, creating social opportunities for people with ID to develop skills to control feelings and emotions and understand social rules can significantly reduce the risk of sexual abuse. The results also show that in addition to the need to provide practical education on personal and menstrual hygiene to women with ID, regular examinations of the reproductive system by obstetricians or midwives, especially in care centers, seem essential.

## Data Availability

The datasets used and/or analyzed during the current study are available from the corresponding. author on reasonable request.
